# Decreasing rates of colectomy for benign neoplasms: A nationwide analysis

**DOI:** 10.1371/journal.pone.0293389

**Published:** 2023-10-25

**Authors:** Sara Sakowitz, Syed Shahyan Bakhtiyar, Saad Mallick, Baran Khoraminejad, Manuel Olmedo, Millicent Croman, Peyman Benharash, Hanjoo Lee

**Affiliations:** 1 Cardiovascular Outcomes Research Laboratories (CORELAB), University of California, Los Angeles, CA, United States of America; 2 Department of Surgery, University of Colorado, Aurora, CO, United States of America; 3 Department of Surgery, University of California, Los Angeles, CA, United States of America; ECU: East Carolina University, UNITED STATES

## Abstract

**Background:**

Despite advances in endoscopic techniques for management of benign colonic neoplasms, a rise in rates of surgical treatment has been reported. We used a nationally representative cohort to characterize temporal trends, patient characteristics, and outcomes associated with colectomy for colonic neoplasms.

**Methods:**

All patients undergoing elective partial colectomy for benign or malignant colonic neoplasms were identified using the 2012–2019 National Inpatient Sample. Those presenting with inflammatory bowel disease, or experiencing intestinal perforation were excluded. Patients with benign neoplasms were classified as the *Benign* cohort (others: *Malignant*). Trends, characteristics, and outcomes were assessed between groups.

**Results:**

Of 569,280 colectomy procedures included for analysis, 153,435 (27.0%) were performed for benign lesions. The proportion of *Benign* operations decreased from 28.6% in 2012 to 23.7% in 2019 (P for trend<0.001). While overall national incidence of colectomy for benign neoplasms decreased from 2012 to 2019 (IRD -1.19, 95%CI -1.20- -1.19), Black patients demonstrated an incremental increase (IRD +0.04, 95%CI +0.02–0.06).

On average, *Benign* was younger (66 [57–72] vs 68 years [58–77], P<0.001), and demonstrated a lower Elixhauser comorbidity index (2 [1–3] vs 3 [2–4], P<0.001), relative to *Malignancy*. Following adjustment, *Benign* demonstrated lower odds of in-hospital mortality (AOR 0.61, 95%CI 0.50–0.74; P<0.001), stoma creation (AOR 0.46, 95%CI 0.43–0.50; P<0.001), and infectious complications (AOR 0.68, 95%CI 0.63–0.73; P<0.001).

**Conclusions:**

The present national study identifies a decrease in colectomy for benign polyps from 2012–2019. Future investigations should identify patients who would most benefit from surgical resection and address persistent inequities in access to screening and treatment for colonic neoplasms.

## Introduction

Colorectal cancer is the second leading cause of cancer-related death with over 150,000 patients diagnosed in the United States each year [1]. Colonoscopy has been repeatedly shown to reduce both the incidence of and mortality from colon cancer, largely through the detection and resection of precancerous polyps [2,3]. However, up to 15% of polyps identified via screening are considered complex and not suitable for polypectomy. Historically, these neoplasms were referred for segmental colectomy [4–6]. Yet, the advent of more sophisticated techniques including endoscopic mucosal resection (EMR), endoscopic submucosal dissection (ESD), and endoscopic full-thickness resection (EFTR) has transformed the landscape of colonic resection and offer potentially safer and less invasive alternatives [7–10]. These techniques have also been demonstrated to be more cost-effective compared to traditional partial colectomy [11,12].

Despite the established efficacy and success of these techniques, prior literature has reported conflicting trends in resection for nonmalignant lesions. Peery *et al*. [13] reported the colectomy rate for nonmalignant lesions was actually increasing. In contrast, Alam *et al*. [6] found declining use of colectomy using a single-institution database. Further, these studies’ datasets included both rectal and anal neoplasms, which would require different management strategies from colonic lesions. Given the burden of adverse events and costs associated with colectomy, a contemporary, comprehensive understanding of trends in strategies to address benign colonic lesions is warranted and could shape future interventions aimed at this cohort.

Thus, using a nationally-representative cohort, the present work sought to characterize trends in colectomy rates for nonmalignant colonic polyps between 2012 and 2019.

## Methods

### Data source and study population

This retrospective cohort study identified all patients (≥ 18 years) undergoing elective partial colectomy for benign or malignant colonic neoplasms in the 2012–2019 National Inpatient Sample (NIS) using previously-reported *International Classification of Diseases*, *Ninth and Tenth Revision* (ICD-9 and ICD-10) procedure codes [13]. Maintained by the Healthcare Cost and Utilization Project (HCUP), the NIS is the largest publicly available national database reporting all-payer hospital discharge information. Using survey-weighted methodology, the NIS samples 20% of patients from all participating hospitals and provides accurately estimates for ~97% of US hospitalizations [14].

### Variable definitions and study outcomes

**Fig 1:**
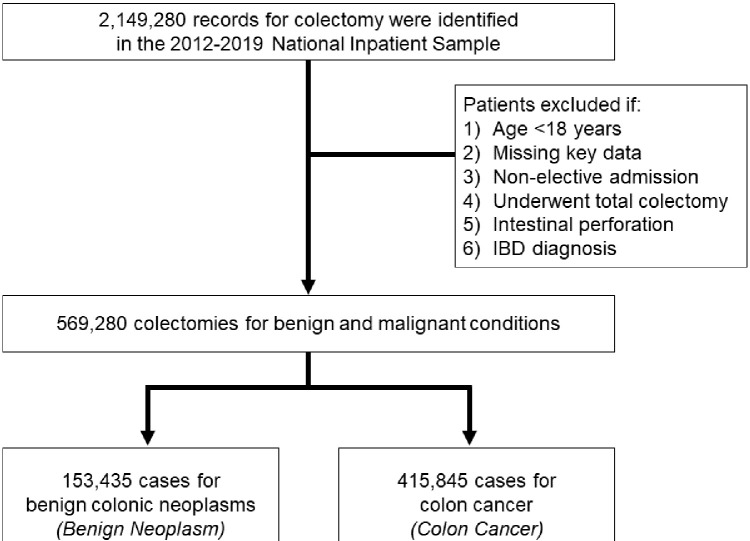
CONSORT diagram with survey-weighted estimates. Of 2,149,280 hospitalizations for colectomy identified in the 2012–2019 NIS, 569,280 patients were included for analysis. Of these, 153,435 (27.0%) were for benign colonic neoplasms. All estimates represent survey-weighted methodology.

The presence of non-malignant colonic polyps or colon cancer was ascertained using relevant ICD-9/10 codes (S1 Table). Those with relevant diagnosis codes for benign neoplasm and without concurrent colon cancer were deemed the *Benign* cohort (others: *Malignancy*). Patient records with codes for both benign and malignant lesions were included in the *Malignancy* cohort. To limit cohort heterogeneity, patients were excluded if they were missing key data (2.7%), underwent total colectomy, experienced intestinal perforation, or presented with inflammatory bowel disease (Fig 1).

Patient, procedure, and hospital characteristics were defined using the HCUP data dictionary [15]. The previously-validated Elixhauser Comorbidity Index was used to assess patient burden of chronic disease at admission [16]. Previously-reported relevant ICD-9/10 codes were used to identify comorbid conditions and perioperative complications, including gastrointestinal complications (bowel ischemia, intestinal perforation, megacolon, hemoperitoneum, and liver complications), stoma creation, respiratory complications (ARDS, respiratory failure, prolonged ventilation, pneumothorax), cardiac complications (cardiac arrest, ventricular tachycardia, ventricular fibrillation, cardiac tamponade, myocardial infarction), stroke complications (cerebral hemorrhagic infarct, transient ischemic attack), thrombotic complications (deep venous thrombosis, pulmonary embolism), procedural complications (accidental puncture, hemorrhage), and infectious complications (bacterial infection, SIRS, sepsis, septicemia, bacteremia, *C*. *difficile*, peritoneal abscess, cellulitis, surgical site infections, ostomy infections, mediastinitis) [17].

Annual center-level colectomy volume was calculated and used to classify hospitals as low-, medium-, or high-volume hospitals following previously reported methodology [18]. Overall hospitalization expenditures were calculated using cost-to-charge ratios provided by HCUP and then inflation-adjusted using the 2019 Personal Healthcare Price Index [19].

### Statistical analysis

Continuous variables are reported as means and standard deviation (SD) if normally distributed or medians and interquartile range (IQR), if non-normally distributed. Categorical variables are reported as frequency (%). The Mann-Whitney *U*, adjusted Wald, and Pearson’s tests were used for bivariate comparison of patient, procedural, and hospital characteristics, as appropriate. Cuzick’s nonparametric test (nptrend) was used to determine significance of temporal trends [20].

Multivariable regression models were generated to consider the independent associations of *Benign* with key outcomes. Model covariates were selected using elastic net regularization, which utilizes a penalized least-squares methodology to minimize collinearity and overfitting [21]. Model outputs are reported as adjusted odds ratios (AOR) if logistic or beta-coefficients (β) if linear, both with 95% confidence intervals (95%CI).

The yearly incidence of colectomy for benign colonic neoplasms was calculated and reported as number of procedures per 100,000 U.S. adults. The total U.S. population was ascertained using publicly-available 2020 U.S. Census data [22]. Age was categorized as 18–49, 50–64, 65–79, and ≥80 years. To compare rates of colectomy, incidence rate differences (IRD) were subsequently computed and expressed as rates per 100,000 adults.

Statistical significance was set at α = 0.05. All statistical analyses were performed using Stata 16.1 (StataCorp, College Station, TX). All data are detailed in accordance with STROBE 2021 reporting standards. Due to the fully deidentified nature of the NIS, this retrospective study was exempted from full review by the Institutional Review Board at the University of California, Los Angeles (IRB #17–001112).

## Results

**Fig 2:**
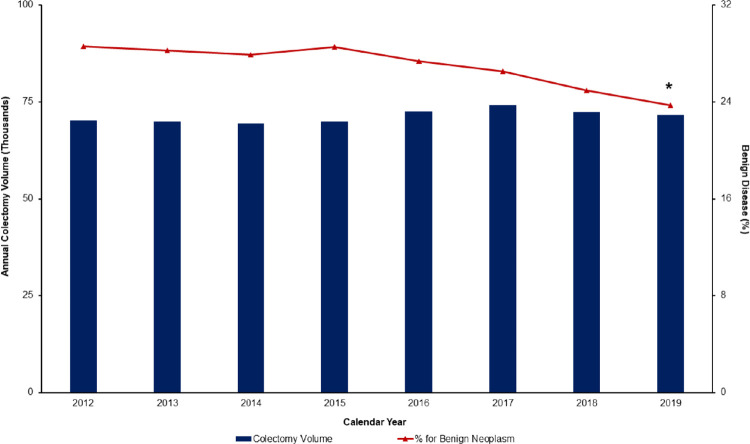
Annual trends of colectomy volume for benign and malignant disease. Both annual colectomy volume and the proportion of procedures for benign colonic neoplasms declined significantly across the study period. * indicates statistical significance, p for trend<0.001.

Of an estimated 569,280 colectomy operations captured during the study period, 153,435 (27.0%) were performed for benign colonic neoplasms. The proportion of procedures for benign disease decreased significantly from 28.6% in 2012 to 23.7% in 2019 (P for trend<0.001) (Fig 2).

**Fig 3:**
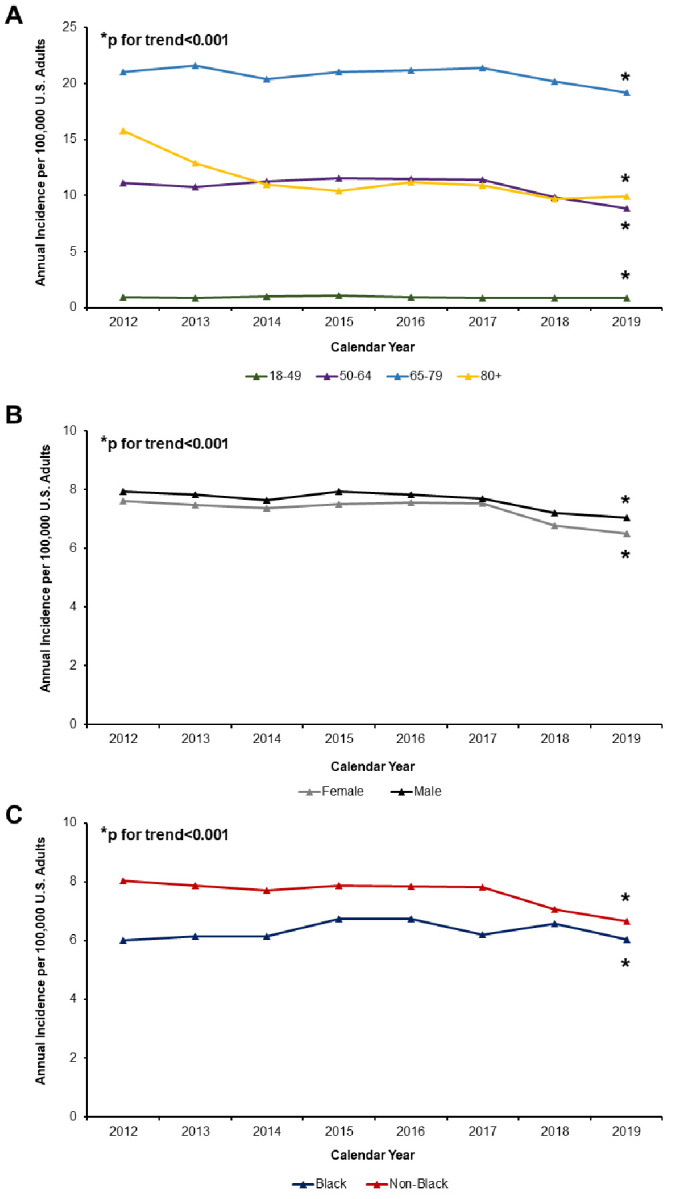
Trends in annual incidence of colectomy for benign neoplasms, stratified by age group. Incidence of colectomy for benign disease decreased significantly from 2012 to 2019 across (A) ages 18–49, 50–64, 65–79, and ≥80 years and (B) sex. While incidence declined among non-Black patients, Black patients demonstrated a small but significant increase in rate of colectomy for benign lesions (C). * indicates statistical significance, p for trend<0.001.

In a sub-group analysis after stratifying the study cohort by age, the national incidence of colectomy for benign neoplasms decreased from 2012 to 2019 (Age 18–49: IRD -0.08, 95%CI -0.08 to -0.08; age 50–64: IRD -2.29, 95%CI -2.32 to -2.27, age 60–79 IRD -1.85, 95%CI -1.94 to -1.76, ≥80 years IRD -5.90, 95%CI -6.08 to -5.71) (Fig 3A). Overall across age strata, national incidence of resection for benign disease decreased from 7.5 in 2012 to 6.3 in 2019 (IRD -1.19, 95%CI -1.20 to -1.19). Rates of colectomy similarly decreased among both males (IRD -0.89, 95%CI -0.90 to -0.88) and females (IRD -1.11, 95%CI -1.12 to -1.10) (Fig 3B). However, when considering Black versus non-Black race, Black patients demonstrated an incremental increase in incidence from 2012 to 2019 (IRD +0.04, 95%CI +0.02 to 0.06). In contrast, the incidence of colectomy for benign lesions decreased among non-Black populations (IRD -1.38, 95%CI -1.39 to -1.38) (Fig 3C).

On average, patients in the *Benign* cohort were younger (66 [57–72] vs 68 years [58–77], P<0.001) and demonstrated a lower Elixhauser comorbidity index (2 [1–3] vs 3 [2–4], P<0.001), relative to *Malignancy*. Further, *Benign* was more frequently White (78.2 vs 76.4%, P<0.001) and privately insured (38.9 vs 34.3%, P<0.001). Compared with *Malignancy*, patients in the *Benign* group less frequently presented with coronary artery disease (6.8 vs 8.2%, P<0.001), renal failure (3.8 vs 5.0%, P<0.001), and anemia (1.5 vs 6.1%, P<0.001) (Table 1).

**Table 1:** Demographic, clinical, and hospital characteristics.

	*Benign* (n = 153,435)	*Colon Cancer* (n = 415,845)	*P-value*
Age (years [IQR])	66 [57–72]	67 [58–77]	<0.001
Female (%)	50.0	49.3	0.07
Elixhauser Comorbidity Index (years [IQR])	2 [1–3]	3 [2–4]	<0.001
*Operative approach (%)*			<0.001
Open	36.6	52.8	
Laparoscopic	63.4	47.2	
*Race/ethnicity (%)*			<0.001
White	78.2	76.4	
Black	11.7	10.3	
Hispanic	6.0	7.1	
Asian/Pacific Islander	1.5	3.2	
Other	2.5	2.9	
*Income quartile (%)*			0.14
>75%	22.6	22.9	
51–75%	25.7	25.1	
26–50%	26.4	26.8	
0–25%	25.3	25.2	
*Insurance coverage (%)*			<0.001
Private	38.9	34.3	
Medicare	52.8	56.3	
Medicaid	5.2	5.9	
Other Payer	3.0	3.5	
*Comorbidities (%)*			
Congestive heart failure	2.5	4.0	<0.001
Coronary artery disease	6.8	8.2	<0.001
Peripheral vascular disease	2.1	2.7	<0.001
Cardiac arrhythmias	6.4	8.9	<0.001
Obesity	10.3	9.7	0.01
Chronic pulmonary disease	8.9	8.3	0.001
Renal failure	3.8	5.0	<0.001
Liver disease	1.6	2.4	<0.001
Anemia	1.5	6.1	<0.001
Electrolyte abnormality	6.0	8.6	<0.001
Coagulopathic disorders	1.2	1.4	0.01
*Annual Hospital Colectomy Volume (%)*			0.10
Lowest tertile	12.5	12.3	
Mid tertile	30.2	29.6	
Highest tertile	57.3	58.1	
*Hospital teaching status (%)*			<0.001
Urban teaching	63.1	65.6	
Urban non-teaching	27.8	24.5	
Rural	9.2	10.0	
*Hospital region (%)*			<0.001
Northeast	16.4	18.6	
Midwest	24.2	24.2	
South	43.3	38.8	
West	16.1	18.4	

Reported as proportions unless otherwise noted. Statistical significance was set at α = 0.05.

*SD, standard deviation.

The *Benign* cohort was more commonly treated in the South (43.3 vs 38.8%), but less often in the Northeast (16.4 vs 18.6%) and West (16.1 vs 18.4%, P<0.001). While *Benign* patients were less likely to receive care at an urban teaching institution (63.1 vs 65.6%, P<0.001), the groups were similarly frequently treated at high-volume institutions (57.3 vs 48.1%, P = 0.10). A sub-group analysis comparing Black to non-Black patients undergoing colectomy for benign disease showed Black patients were more commonly in the lowest income quartile (48.1 vs 22.5%, P<0.001) and received care in the South (63.4 vs 40.8%, P<0.001). As compared to non-Black, Black patients were more often insured by Medicaid (9.3 vs 4.7%, P<0.001) (S2 Table).

Relative to *Malignancy*, *Benign* less frequently experienced in-hospital mortality (0.4 vs 0.8%, P<0.001), gastrointestinal complications (0.3 vs 0.4%, P = 0.05), and stoma creation (2.9 vs 6.3%, P<0.001). The cohorts were similar in rates of intraoperative complications (2.0 vs 2.0%, P = 0.99), but *Malignancy* more often faced infectious complications (3.0 vs 4.5%, P<0.001). In addition, *Benign* experienced shorter duration of hospitalization (LOS) (4 [3–5] vs 5 days [3–7], P<0.001) and lower hospitalization expenditures ($13,700 10,400–18,700] vs 16,400 [$12,300–23,300], P<0.001).

After risk adjustment, *Benign* demonstrated lower odds of in-hospital mortality (AOR 0.61, 95%CI 0.50–0.75; P<0.001), stoma creation (AOR 0.46, 95%CI 0.43–0.50; P<0.001), and infectious complications (AOR 0.68, 95%CI 0.63–0.73; P<0.001). However, the two cohorts experienced similar likelihood of gastrointestinal (AOR 0.90, 95%CI 0.71–1.14; P = 0.39) and intraoperative complications (AOR 1.05, 95%CI 0.95–1.16; P = 0.35). Further, *Benign* was linked with a 1-day decrement in LOS (95%CI -1.1 to -0.90 days; P<0.001) and a $3,600 reduction in hospitalization expenditures (95%CI -$3,800 to -3,300) (Table 2).

**Table 2:** Unadjusted and adjusted outcomes of *Benign* as compared to *Malignancy*.

	Unadjusted			Adjusted		
	*Benign*	*Malignancy*	*P-Value*	*Benign*	*95% CI*	*P-Value*
**Clinical outcomes**						
In-hospital mortality	0.4	0.8	<0.001	0.61	0.50–0.75	<0.001
Gastrointestinal complications	0.3	0.4	0.05	0.90	0.71–1.14	0.39
Stoma creation	2.9	6.3	<0.001	0.46	0.43–0.50	<0.001
Intraoperative complications	2.0	2.0	0.99	1.05	0.95–1.16	0.35
Infectious complications	3.0	4.5	<0.001	0.68	0.63–0.73	<0.001
Respiratory complications	2.6	4.1	<0.001	0.71	0.65–0.77	<0.001
Blood transfusion	<0.1	<0.1	0.39	0.86	0.39–1.89	0.71
Cardiac complications	0.9	1.5	<0.001	0.71	0.61–0.81	<0.001
Thrombotic complication	0.3	1.0	<0.001	0.38	0.31–0.47	<0.001
Stroke complications	0.2	0.3	<0.001	0.64	0.46–0.89	0.01
Renal complications	3.9	5.6	<0.001	0.78	0.73–0.84	<0.001
Non-home discharge	4.2	9.9	<0.001	0.44	0.41–0.47	<0.001
**Resource utilization**						
Length of stay (days) [IQR]	4 [3–5]	5 [3–7]	<0.001	-1.0	-(1.1–0.9)	<0.001
Cost (USD $1,000) [IQR]	13.7 [10.4–18.7]	16.4 [12.3–23.3]	<0.001	-3.6	-(3.8–3.3)	<0.001

Outcomes reported as proportions or as Adjusted Odds Ratio (AOR) with 95% confidence intervals (95% CI).

*IQR, interquartile range; *USD*, United States dollar.

## Discussion

While complex colonic polyps have traditionally required surgical removal of the involved colonic segment, advances in endoscopic techniques have transformed their management. In contrast with recent studies, we report an overall decline in the national incidence of colectomy for benign indications over the last decade. Further, we identified continued racial disparities in management of benign colonic polyps, with Black patients more likely to undergo surgical resection. Compared to colectomy for malignant neoplasm, resections for benign lesions appear associated with reduced in-hospital mortality and markers of resource use. Several of these findings merit further discussion.

In this nationally-representative study, we reported a declining incidence of segmental colectomy for benign colonic neoplasms from 2012 to 2019 across all age groups. This finding contradicts work by Peery *et al*. [13] who demonstrated increased incidence of colorectal resection procedures for nonmalignant polyps from 2010 to 2014. Large population studies have shown that while fecal immunochemical tests (FIT) and multi-target stool assays may be acceptable screening tools, colonoscopy yields higher rate of adenoma detection [23,24]. Increased adoption of these screening tools may lead to reduced number of colonoscopies and subsequent colectomies for benign neoplasms. Future studies, such as the ongoing Voyage trial, [25,26] can offer additional insight regarding the potential for these noninvasive assays to further replace screening colonoscopy. However, given no significant change in the national rates of screening colonoscopy over the last decade [13,27,28], we hypothesize the decline in colectomy incidence in this study may stem, at least in part, from increased adoption of endoscopic techniques for removal of benign lesions such as EMR, ESD and EFTR. Briefly, EMR with submucosal lift has been established as a safe and effective modality for removal of superficial, sessile or flat lesions <20mm in size [29–31]. For lesions ≥ 20mm not amenable to en bloc resection or those involving upper 1/3 of the submucosa, ESD has emerged as an promising new modality [32–38]. Lastly, for difficult-to-resect neoplasms, subepithelial tumors, or lesions with dense fibrosis, EFTR emerged as a new addition to the armamentarium of endoscopic management which still offers a less-invasive alternative to partial colectomy [8,9,39,40]. To date, significant evidence has shown decreased postoperative complications and costs associated with these endoscopic treatment modalities, as compared with surgical resection [13,29,41]. However, while these methods have been broadly adopted in East Asian countries, implementation in Europe and the United States has been more gradual [7,42,43]. This more limited adoption has been attributed to procedural complexity, lack of standardized training, and absence of distinct Current Procedural Terminology codes that would permit center reimbursement [7,40,44]. Thus, structured educational and reimbursement pathways need to be implemented to encourage institutional adoption and dissemination of these endoscopic techniques, with significant potential clinical impact.

Notably, the present study found that Black patients demonstrated a small but significant increase in rates of colectomy for benign neoplasms from 2012 to 2019. Our sub-group analysis also revealed these patients to be predominantly of lowest-quartile income, insured by Medicare or Medicaid, and located in the South. Potentially living in medically under-served areas with reduced availability of gastroenterologists or endoscopy facilities [45], these patients may have less access to advanced, minimally-invasive FIT or stool testing modalities, as well as non-surgical interventions. Additionally, previous work has identified lower use of screening and surveillance colonoscopies among Black patients [46,47], which may preclude endoscopic removal of benign neoplasms [48]. Ultimately, given continued disparities in screening, treatment, and referral to advanced interventions, new programs and policies are needed to broaden access to and engagement with care for benign colonic neoplasms, particularly among under-resourced populations. Indeed, prospective interventions could consider expanding access to noninvasive colon cancer screening modalities, establishing referral pathways to expand access to advanced endoscopy, and improving both patient and clinician knowledge of non-surgical interventions.

Our subgroup analysis of patients over 80 years revealed the largest decrease in incidence of colectomy for benign lesions over the study period. While the reasons underlying these trends are yet to be fully elucidated, specific changes in management of geriatric patients may play an important role. Recent work has demonstrated the safety and efficacy of endoscopic mucosal resection among this population [49,50]. Further, given the known complications of colectomy, higher surgical risk profile, and rapidly growing cohort of elderly patients, these patients may be referred at greater frequency for endoscopic intervention [51]. Considering the aging U.S. population, future work is needed to consider optimal surgical or endoscopic management among these patients.

Not surprisingly, the mortality rates following segmental colectomy remained under 1% among both the *Benign* and *Malignant* cohorts, demonstrating the relative safety of the procedure. However, relative to *Malignant*, *Benign* patients experienced decreased perioperative complications, non-home discharge, and resource utilization. While surgical resection for *Benign* was linked with decreased expenditures relative to *Malignant*, the median cost per-hospitalization for segmental colectomy ($14,000) vastly outweighs the estimated $5,500–6,500 cost for endoscopic resection [11,12]. Beyond the cost-effectiveness of endoscopy in the short-term, though, colectomy could also be linked with significant added long-term expenditures. Similar to other studies, we found 2.9% of *Benign* patients to require an ostomy. Notably, ostomy care can require significant time and financial involvement, with potential for skin breakdown, stomal stenosis, prolapse, hernia formation, and bleeding, among other complications [52]. In addition, ostomy reversal has been linked with significant complications and postoperative mortality [53,54]. Thus, while we report safety of colectomy in the acute period, the procedure can have significant implications for long-term health and quality of life.

This study has several limitations. We used the NIS, an administrative database that relies on administrative *International Classification of Diseases* coding. While the codes utilized in this study were previously-validated [13], coding practices can vary at the provider, hospital, and regional level. Granular patient data was unavailable for analysis, including imaging, laboratory, or physiologic data. We could not access information regarding fecal immunohistochemistry histochemistry tests, fecal occult blood testing, or multi-target stool assays. Given our study was limited to in-patient records, we could not assess outpatient colonoscopy or surgical endoscopy data. Further, specific colonoscopy findings including polyp size and morphology were not documented, but may have shaped clinician judgment in referring patients for colectomy. We additionally could not assess individual patient colonoscopy or disease history, and could not determine whether patients were offered endoscopic management as an alternative option to colectomy. To limit heterogeneity of our cohort, we excluded patients with inflammatory bowel disease and exclusively evaluated those undergoing segmental colectomy with a concurrent diagnosis code for colonic, and not rectal, lesions. However, these factors may have influenced our estimations of national incidence. Despite these limitations, our study utilized robust statistical methodology and the largest all-payer national database to perform a contemporary analysis of incidence of colectomy for benign colonic lesions.

## Conclusion

In conclusion, surgical resection for benign colonic polyps is decreasing at the national level. These findings are consistent across age and sex stratifications, and concur with a growing body of work supporting the safety, efficacy, and reduced morbidity of endoscopic resection as an alternative management strategy. However, Black patients demonstrated an increase in incidence of colectomy, suggesting disparities persist in access to and engagement with minimally invasive endoscopic techniques for benign lesions. Our study calls for future work to identify patients who would most benefit from surgical versus endoscopic resection and address persistent inequities in screening, surveillance, and care for colonic neoplasms.

## Supporting information

S1 Table ICD-9/10 codes for disease indication. (DOCX)

S2 Table Demographic, clinical, and hospital characteristics of Black vs. Non-Black patients undergoing colectomy for benign colonic neoplasms. Reported as proportions unless otherwise noted. Statistical significance was set at α = 0.05. *IQR, interquartile range.
(DOCX)
