# Supplementary material for: Decreasing rates of colectomy for benign neoplasms: A nationwide analysis
Source: PLoS One. 2023 Oct 25;18(10):e0293389. doi: 10.1371/journal.pone.0293389 (PMC10599571; doi:10.1371/journal.pone.0293389)
Supplement: S1 Table — (DOCX) [file pone.0293389.s001.docx]

**Supplemental Table S1:**

TITLE: ICD-9/10 Codes for Disease Indication.

|  | **ICD-9/10 Code** |
| --- | --- |
| Benign colonic neoplasm | 211.3, D120, D121, D122, D123, D124, D125, D126, D127 |
| Colon cancer | 153, 154, 230.3, 230.4, C18, C19, D01.0, D01.1, C7A.021, C7A.022, C7A.023, C7A.024, C7A.25, C7A.029, C7A.096, C7A.1, C7A.8 |
