# Supplementary material for: Decreasing rates of colectomy for benign neoplasms: A nationwide analysis
Source: PLoS One. 2023 Oct 25;18(10):e0293389. doi: 10.1371/journal.pone.0293389 (PMC10599571; doi:10.1371/journal.pone.0293389)
Supplement: S2 Table — Reported as proportions unless otherwise noted. Statistical significance was set at α = 0.05. *IQR, interquartile range. (DOCX) [file pone.0293389.s002.docx]

**Supplemental Table S2:**

TITLE: Demographic, clinical, and hospital characteristics of Black vs. Non-Black patients undergoing colectomy for benign colonic neoplasms.

CAPTION: Reported as proportions unless otherwise noted. Statistical significance was set at α= 0.05.

**IQR*, interquartile range

|  | ***Non-Black***  (n = 136,150) | ***Black***  (n = 17,250) | ***P-value*** |
| --- | --- | --- | --- |
| Age (years [IQR]) | 66 [57-73] | 64 [56-71] | <0.001 |
| Female (%) | 49.1 | 56.5 | <0.001 |
| Elixhauser Comorbidity Index (years [IQR]) | 2 [1-3] | 2 [1-3] | <0.001 |
| *Operative approach (%)* |  |  | 0.10 |
| Open | 36.4 | 37.9 |  |
| Laparoscopic | 63.6 | 62.1 |  |
| *Income quartile (%)* |  |  | <0.001 |
| >75% | 23.9 | 12.4 |  |
| 51-75% | 26.7 | 17.8 |  |
| 26-50% | 27.0 | 21.7 |  |
| 0-25% | 22.5 | 48.1 |  |
| *Insurance coverage (%)* |  |  | <0.001 |
| Private | 39.0 | 38.2 |  |
| Medicare | 53.4 | 48.2 |  |
| Medicaid | 4.7 | 9.3 |  |
| Other Payer | 2.9 | 4.3 |  |
| *Hospital region (%)* |  |  | <0.001 |
| Northeast | 16.8 | 13.3 |  |
| Midwest | 25.2 | 16.8 |  |
| South | 40.8 | 63.4 |  |
| West | 17.3 | 6.4 |  |
